# Metabolic-endocrine disruption due to preterm birth impacts growth, body composition, and neonatal outcome

**DOI:** 10.1038/s41390-021-01566-8

**Published:** 2021-05-26

**Authors:** Lea Sophie Möllers, Efrah I. Yousuf, Constanze Hamatschek, Katherine M. Morrison, Michael Hermanussen, Christoph Fusch, Niels Rochow

**Affiliations:** 1 Department of Pediatrics, Paracelsus Medical University, Nuremberg, Germany; 2 Department of Pediatrics, McMaster University, Hamilton, ON Canada; 3 Aschauhof, Eckernförde-Altenhof, Germany; 4 Department of Pediatrics, University Medical Center Rostock, Rostock, Germany

## Abstract

**Abstract:**

Despite optimized nutrition, preterm-born infants grow slowly and tend to over-accrete body fat. We hypothesize that the premature dissociation of the maternal–placental–fetal unit disrupts the maintenance of physiological endocrine function in the fetus, which has severe consequences for postnatal development. This review highlights the endocrine interactions of the maternal–placental–fetal unit and the early perinatal period in both preterm and term infants. We report on hormonal levels (including tissue, thyroid, adrenal, pancreatic, pituitary, and placental hormones) and nutritional supply and their impact on infant body composition. The data suggest that the premature dissociation of the maternal–placental–fetal unit leads to a clinical picture similar to panhypopituitarism. Further, we describe how the premature withdrawal of the maternal–placental unit, neonatal morbidities, and perinatal stress can cause differences in the levels of growth-promoting hormones, particularly insulin-like growth factors (IGF). In combination with the endocrine disruption that occurs following dissociation of the maternal–placental–fetal unit, the premature adaptation to the extrauterine environment leads to early and fast accretion of fat mass in an immature body. In addition, we report on interventional studies that have aimed to compensate for hormonal deficiencies in infants born preterm through IGF therapy, resulting in improved neonatal morbidity and growth.

**Impact:**

- Preterm birth prematurely dissociates the maternal–placental–fetal unit and disrupts the metabolic-endocrine maintenance of the immature fetus with serious consequences for growth, body composition, and neonatal outcomes.
- The preterm metabolic-endocrine disruption induces symptoms resembling anterior pituitary failure (panhypopituitarism) with low levels of IGF-1, excessive postnatal fat mass accretion, poor longitudinal growth, and failure to thrive.
- Appropriate gestational age-adapted nutrition alone seems insufficient for the achievement of optimal growth of preterm infants.
- Preliminary results from interventional studies show promising effects of early IGF-1 supplementation on postnatal development and neonatal outcomes.

## Introduction

The American Academy of Pediatrics has proposed that preterm infants should achieve the “…rate of growth and composition of weight gain for a normal fetus of the same postmenstrual age… .”^1^ These infants are expected to mimic fetal growth rates of their term-born counterparts with comparable body composition and functional outcomes.^1^ However, at term age, the body composition of preterm-born infants differs from term-born infants. They are generally shorter in length, which suggests slower growth, and have higher body fat percentages, greater fat mass, and lower lean mass compared to term-born infants.^2,3^ These findings are alarming in light of the concept of Developmental Origin of Health and Disease (DOHaD), which links unfavorable early-life body composition outcomes with adverse long-term health outcomes.^4,5^

Recent breakthroughs in perinatal medicine have readjusted the focus of neonatologists from solely increasing the survival rates of preterm infants, towards improving nutrition, growth, and development. Achieving the recommended nutritional intake enables preterm-born infants to reach or even to exceed term-born weight trajectories,^1,6,7^ which may allow the lean body mass of preterm infants at term age to match the levels of term-born neonates.^7^ However, the fat mass levels of preterm infants at term age often surpass their term-born counterparts, reaching levels comparable to that of 2–3-month-old term-born infants.^2,3,5,8,9^ To the best of our knowledge, no study to date has reported similar fat mass and lean mass in preterm- and term-born infants with the same weight at term age.

During pregnancy, the maternal–placental unit supplies the fetus with a tightly regulated metabolic and endocrine network.^10^ It serves as the fetal lung, provides nutrients for the growing fetus, and controls endocrine and immunological functions, as well as thermogenesis. At term, the fetus has sufficiently matured so that the disconnection from the maternal–placental unit occurs without difficulties. Mature newborns readily adapt to extrauterine conditions. Conversely, preterm birth prematurely dissociates the maternal–placental–fetal unit and thereby disrupts the metabolic-endocrine supply of the immature neonate. This could result in serious cardio-pulmonary, metabolic, and endocrine deficiencies. While acute life support in neonatal intensive care is provided for respiration (mechanical ventilation), circulation (inotropes and fluids), and nutrition (parenteral nutrients), the acute metabolic-endocrine disruption could remain unrecognized and is therefore often untreated.

The aim of this review is to outline the essentials of physiological intrauterine metabolic-endocrine regulation. We summarize the effects of intact metabolic-endocrine regulation on postnatal fetal growth and body composition in term infants. Further, we highlight the metabolic effects of the premature dissociation of the maternal–placental–fetal unit in preterm birth and compare lean and fat mass accretion in preterm and term infants. Finally, we review clinical trials that explore the use of hormone supplementation therapies for the immature neonate.

### Overview of hormones involved in growth

**Fig. 1:**
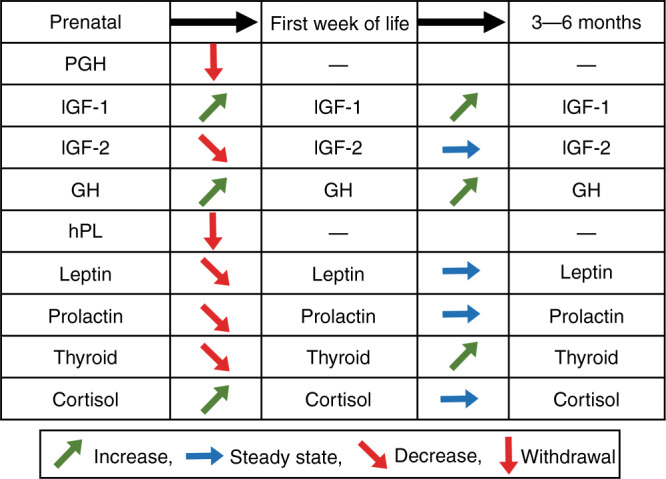
Change in hormonal activity during the transition from fetal to postnatal life. Hormonal activity levels in the newborn undergo changes, caused by the dissociation of the maternal-placental-fetal unit with birth and the maturation of the newborn. PGH placental growth hormone, IGF insulin-like growth factor, GH growth hormone, hPL human placental lactogen, Thyroid thyroid hormones.

The maternal–placental–fetal unit secretes a variety of hormones that influence intrauterine growth. The main hormones and growth factors released by the placenta that contribute to fetal development are placental growth hormone (PGH), human placental lactogen (hPL), insulin-like growth factor 2 (IGF-2), and corticotropin-releasing hormone (CRH).^11,12^ In response to hormonal or nutritional stimuli, maternal insulin-like growth factor 1 (IGF-1), leptin, insulin, and thyroid hormones are released and stimulate fetal growth directly and indirectly. In the fetus, sufficient substrate availability then triggers the release of IGF-1, IGF-2, insulin, and leptin, which directly stimulate growth-promoting processes. At birth, the newborn is removed from the maternal–placental supply, resulting in changes in hormonal levels (Fig. 1). Postnatally, infant growth is regulated by IGF-1, IGF-2, insulin, leptin, and thyroid hormones until maturation of the growth hormone (GH)-regulated pathway occurs and the GH-axis becomes the primary regulator of growth.^10,11,13–17^ To analyze the interactions of maternal, fetal, placental, and postnatal hormones, the characteristics and functions of selective hormones are discussed in the following paragraphs.

### Placental growth hormone

PGH is produced by the syncytiotrophoblast and has an impact on the release of maternal hormones that act on fetal growth, as shown by its positive association with birth weight in many studies.^18–20^ PGH mediates anabolic effects (somatotrophic, lactogenic, and lipolytic properties) on the body by binding to the GH receptor (GHR), a type I cytokine receptor.^18^ During pregnancy, PGH suppresses the release of pituitary-derived maternal GH, making maternal GH undetectable in blood by 15 weeks of gestation until it reemerges after placental delivery.^21^ Furthermore, PGH takes over the regulation of maternal IGFs and directly stimulates the production of IGFs in maternal tissue.^18,21^ PGH is released directly into the maternal circulation, where it can be detected as early as 5 weeks of gestation, and continuously increases, reaching a mid-gestational peak of 20–40 ng/mL at 34 weeks.^18,21,22^ PGH release responds positively to maternal glucose levels in order to increase insulin resistance for a greater glucose gradient and delivery to the fetus.^18^ It exerts its biological actions exclusively on maternal and uteroplacental tissues, increasing maternal IGF-1 production, enhancing maternal substrate production (maternal fat accumulation), availability, and nutrient delivery to the fetus.^18^ Although PGH predominantly acts on the mother to create a growth-promoting environment for the fetus, a recent study detected PGH in fetal blood, demonstrating that a direct release or active transport of PGH into the fetal circulation is possible.^18,23^

### Human placental lactogen

The syncytiotrophoblast produces hPL. hPL levels rise progressively with the length of gestation and correlate with placental mass.^21^ It is released directly into the maternal circulation at significant levels, reaching a peak of 5000–7000 ng/mL at 32–35 weeks gestation, and is also directly released into the fetal circulation, reaching lower levels of 20–50 ng/mL at term.^21^ hPL binds with low affinity to the GHR and with high affinity to the prolactin receptors in maternal tissues and the placenta, as well as those widespread throughout the fetus during the first and second trimester.^23^ In the mother, hPL stimulates an increase in IGF-1 levels and affects metabolism by inducing lipolysis and increasing free fatty acid utilization, which spares glucose for the fetus.^23^ Consequently, hPL increases the availability and transport of substrates and enhances placental growth and function, indirectly contributing to fetal growth.^11,21,23^ Furthermore, it counteracts the metabolic stress caused by PGH-mediated insulin resistance, decreasing the risk of gestational diabetes mellitus through pancreatic adaptation, such as upregulation of maternal beta-cell replication, beta-cell mass expansion, and insulin production.^21^ In addition, hPL stimulates the production of insulin and IGFs in the fetus, directly promoting IGF-mediated growth, especially tissue differentiation and growth.^11,23^

### Estrogen and progesterone

Estrogen and progesterone have less impact on early fetal growth compared to other growth-promoting factors. They are produced by the syncytiotrophoblast and work concurrently with placental hormones (PGH and hPL) and maternal IGF to maintain the pregnancy and create a growth-permitting environment.^11^ During pregnancy, materno-placental estrogen increases uteroplacental blood flow and stimulates the production of maternal thyroid hormones. Increasing estrogen levels in the third trimester also stimulate fetal prolactin production, which in turn promotes fetal fat mass accretion.^24^ Progesterone contributes to increased food intake which precedes a rise in maternal leptin levels and suppresses maternal cell-mediated immunity to prevent rejection of the fetus.^21^

### Insulin

The peptide hormone insulin is produced by the beta cells of the pancreas in response to increased blood glucose levels. It acts as a signal for nutrient availability.^11^ Insulin causes the assimilation of glucose and amino acids into cells, functioning as a major growth promoter in utero by stimulating fat storage, tissue accretion, glycogen synthesis, and inhibiting fat breakdown.^25,26^ It regulates the production of leptin and stimulates fetal IGF-1 production, which occurs mainly in the liver and multiple peripheral tissues until the newborn’s GH-axis is matured and begins to take over growth regulation by 6–9 months of life.^14,27,28^ During pregnancy, insulin is not transferred across the placenta. Instead, fetal insulin levels increase and promote growth in response to maternal glucose transfer.^20^ High fetal insulin in response to maternal hyperglycemia is responsible for the increased growth and macrosomia observed in infants born to mothers with gestational diabetes.^29^ It also increases the risk for postnatal hypoglycemia in the newborn. In contrast, babies with pancreatic agenesis or mutations in the insulin receptor gene show findings of severe intrauterine growth retardation. This demonstrates the integral role of insulin in normal intrauterine growth and development.^26^

### Insulin-like growth factors (IGF-1 and IGF-2)

IGF-1 and IGF-2 are among the most important hormones involved in growth.^30^ IGFs are released by the liver, uterus, and peripheral tissues in response to environmental signals such as nutrients, oxygen, and hormones. In the placental tissue, IGF-2 can be found throughout the pregnancy, while IGF-1 appears in low abundance in the second and third trimester.^31^ IGFs modulate local growth through induction of cell proliferation, differentiation, and DNA synthesis by binding to the type I IGF receptor and insulin receptor, which leads to the activation of the Ras/Raf/MAP kinase pathway through the receptor tyrosine kinase.^20,32–35^ Furthermore, they increase glucose and amino acid uptake while simultaneously inhibiting protein breakdown as IGF stimulates the upregulation of tissue glucose transporter abundance.^10,36,37^ During pregnancy, maternal IGFs influence fetal growth indirectly as they do not cross the placenta in significant quantities. Instead, maternal IGFs affect the transport of glucose and amino acids, as well as placental function.^11,20,38^ IGF-2 is produced by the placenta and directly released into the maternal and fetal circulation and regulates fetal growth.^31^

While IGF-1 predominantly affects postnatal growth, IGF-2 is released in greater quantities during fetal life.^30,32^ The growth-promoting actions of IGF-2 on the fetus may be indirect and mediated through changes in placental growth and nutrient transport.^34^ Fetal IGF concentrations are also affected by insulin, thyroxine (T4), and glucocorticoids.^34^ The strong correlation of IGF-1 and IGF-2 with birth weight and postnatal weight gain has been described in many studies.^10,11,27,35,39^ Studies have shown that a deletion of the IGF-1 receptor can lead to severe growth restriction. Conversely, an abundance of IGF-2 leads to macrosomia and generalized organomegaly in infants.^34^ Although pituitary-derived GH is the main regulator of IGF synthesis in humans, during pregnancy, the regulation of maternal IGFs is controlled by PGH.^35^ In the fetus and newborn, nutrition increases the insulin-stimulated production of IGFs, serving as the main regulator of growth until the GH-axis has matured.^14,15,27,28,40^

### Insulin-like growth factor-binding proteins (IGFBPs)

IGFBPs affect the bioavailability of IGFs. IGFBPs bind IGFs with higher affinities than their receptors, therefore segregating IGFs from the receptors. Through the action of IGFBPs, transport of IGFs is enabled, and the half-life of IGFBPs-bound IGFs is increased.^24,41^ At least six different types of IGFBPs have been identified in human tissues. The most prevalent types of IGFBPs being IGFBP-1 to -4.^34^ IGFBP-3 is the principal carrier of IGF-1 and IGF-2 in serum, binding 80–95% of free IGF-1, creating a reservoir in the circulation and regulating its bioavailability.^11,42^ While IGFBP-1 and -2 inhibit IGF action, IGFBP-3 potentiates its effect and correlates positively with growth.^28,39,43^ Further, it was shown that the nutrient intake of preterm infants affected IGFBP levels. A higher energy intake was positively correlated with IGFBP-3 and protein intake was negatively related to IGFBP-2 in preterm infants.^44^ Data on the role of IGFBPs during pregnancy is very limited.

### Leptin

Leptin is an adipocyte-derived satiety factor.^15^ It is generally produced by adipose tissue and by the placenta and binds to leptin receptors in the brain, peripheral tissues (lung, kidney, adipocytes, endothelial cells, blood cells, stomach, muscle, liver, pancreatic islets, osteoblast, endometrium, placenta, and umbilical cord), and immune cells. Furthermore, it has direct effects on many cell types throughout the body.^45^ Placental leptin manages placental functions in an autocrine and paracrine manner, as it modulates proliferation, protein synthesis, invasion, and apoptosis of placental cells. The deregulation of leptin levels, therefore, has been correlated with the pathogenesis of various disorders associated with gestation, including gestational diabetes, pre-eclampsia, and intrauterine growth restriction.^45^ Most of the placental leptin is released into the maternal circulation. In contrast, the total placental leptin released into the fetal circulation is higher than the proportion of the classical placental hormones such as human chorionic gonadotropin (hCG) and hPL.^46^ Leptin concentrations correlate positively with fat mass and function as a marker for substrate availability.^16,17,35^ Leptin is also known to affect energy expenditure and body composition by signaling through hypothalamic receptors.^17,47^ Higher leptin levels are positively correlated with resting energy expenditure, stimulating the metabolic rate and disfavoring hibernation.^46,48^ The mediation of insulin’s anabolic actions, its interleukin-6 like proinflammatory qualities, and the stimulation of bone growth contribute to the growth-promoting effect of leptin in utero.^26,48,49^ Leptin has emerged as an important modulator of immune function and is considered to be a critical link between energy balance and host defense responses to pathogens.^50^

Postnatally, leptin also has an effect on the pituitary GH production, as low leptin levels can cause a decrease in GH levels.^51^

### Prolactin

The hormone prolactin is produced by the fetal anterior pituitary gland.^52^ Levels increase by 25–30 weeks of gestation.^24^ The increase in fetal prolactin is stimulated by a rise in maternal estrogen levels. Prolactin does not cross the placenta.^52^ Prolactin and the abundance of the prolactin receptor play a significant role in organ and tissue development, especially in the differentiation and maturation of white and brown adipose tissue.^52,53^ Fetal prolactin binds to its receptors, which are located in multiple tissues, and together with IGF-2 it enhances fat mass and adipocyte number in the fetus. Peak levels of prolactin are reached at 30 weeks, which indicates that it supports fetal fat mass accretion during the third trimester.^24,54^

### Glucocorticoids

Cortisol, being the most important glucocorticoid, plays a role in fetal maturation and growth. It is produced by the adrenal cortex in response to hypothalamic and placental CRH and binds to glucocorticoid receptors, which are especially abundant in mineralocorticoid target tissues (kidney, colon, adrenal, and placenta).^11,12,55^ An increase in cortisol levels is seen in states of stress and undernutrition, such as protein restriction. Cortisol generally has growth inhibitory qualities: IGF and leptin levels decrease in response to its release, and pituitary GH secretion in infants is suppressed.^11,16,56^ In addition, weight gain, linear growth, and head circumference are inhibited.^57^ Infants exposed to great levels of cortisol during gestation show a significant reduction in birth weight.^11^ The enzymes 11 beta-hydroxysteroid dehydrogenases 1 and 2 control the passage of maternal cortisol to the fetus during pregnancy, by converting active cortisol into inactive cortisone.^11,56^ In the fetus, cortisol shifts mass accretion to tissue and organ maturation.^11,37^ This makes sense physiologically, as inhibition of IGF-1 secretion allows the available energy resources to be used for maturation instead of mass accretion.

### Thyroid hormones

The thyroid hormones triiodothyronine (T3) and T4 play a critical role in the normal development of the fetus and infant, especially in the maturation of the brain, which has been shown in observational studies.^58^ They regulate the overall metabolic rate, stimulate normal growth through effects on IGF-1 levels, contribute to the maturation of the central nervous system, and have further effects on heart, bone, and muscle development. It has been shown that hypothyroidism can lead to changes in IGF-1 gene expression and subsequent reductions in the levels of circulating IGFs, which can lead to fetal growth retardation.^34^ In pregnancy, the maternal production of T3 and T4 is stimulated by placental estrogen and hCG. Maternal thyroid hormones are partially permeable across the placenta. This transfer of maternal thyroid hormones to the fetus, in combination with fetal production of thyroid hormones by 20 weeks postmenstrual age (PMA), is critical for early fetal development and may be neuroprotective for a fetus with hypothyroidism.^13,58^ In addition, thyroid hormones are required for optimal postnatal growth.^58^

### Growth hormone

GH is secreted in the anterior pituitary gland by somatotropic cells in a pulsatile manner in response to GH-releasing hormone, acting as the main regulator of IGF production in humans. It acts by binding to a specific GHR (type I cytokine receptor) and prolactin receptor on the plasma membrane of target cells.^18^ GH also directly binds to its receptors in the growth plate.^26^ It has metabolic and growth-promoting effects, which include directly stimulating growth at the epiphyseal growth plate and promoting lipolysis, linear bone growth, and the accretion of lean mass.^35^ However, GH action is inhibited by PGH during pregnancy. In newborns, particularly in preterm infants, the immaturity of GHRs renders the growth-promoting effect insignificant until maturation of the GH-axis at 6–9 months of life.^14,24,59,60^ In addition to the GH-releasing hormone, factors affecting GH production are nutrition, leptin, and somatostatin, which inhibits GH secretion.^26,61^

### Hormonal influence on growth in utero

**Fig. 2:**
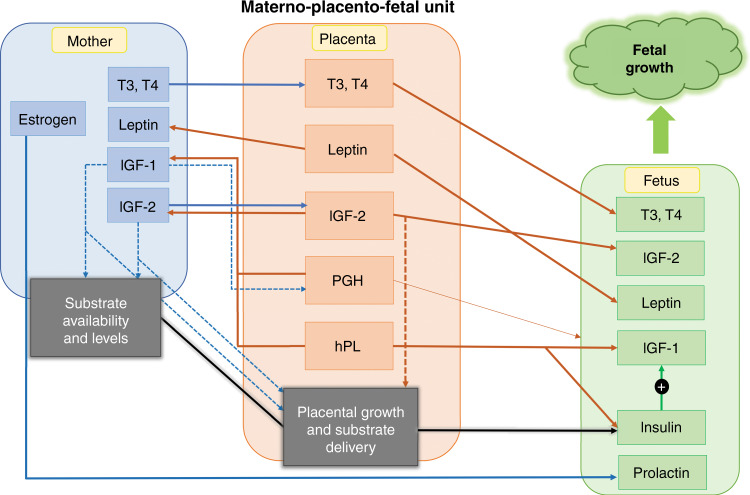
Regulation of fetal growth control. Fetal growth is regulated by hormonal and nutritional interactions of the maternal-placental-fetal unit. Full lines illustrate the transfer of hormones or nutrients, dotted lines represent the stimulating effect on targets, and the thickness of lines correlates with the intensity of the effect; PGH placental growth hormones, hPL human placental lactogen, IGF insulin-like growth factor, T3  triiodothyronine, T4 thyroxine.

Significant associations between fetal weight and circulating levels of placentally produced hormones have been reported.^11,18,35^ Placental hormones promote fetal growth by increasing substrate availability in the mother, improving substrate transport to the fetus, and directly stimulating maternal and fetal IGF-1 production (Fig. 2). In addition, the placenta releases IGF-2 into the fetal circulation, directly affecting fetal growth.^20^ Since glucose serves as the most important nutrient for fetal growth, multiple mechanisms are in place to secure sufficient food intake, energy storage, and nutrient availability.^11^

### Increased substrate availability

PGH acts as an antagonist to maternal insulin by impairing maternal glucose utilization to increase circulating blood glucose levels for fetal substrate availability. With the mid-gestational peak of PGH levels, maternal insulin resistance occurs.^18,21,62^ Maternal insulin resistance, even if it puts metabolic stress on the mother, creates a nutrient gradient, allowing the mother to spare glucose and amino acids for placental growth, thereby increasing the nutrient transport to the fetus.^21,63^ As the placenta has insulin receptors, maternal insulin can enhance placental function and placental nutrient transport to the fetus through effects on angiogenesis and vasculogenesis.^64^ Multiple studies have found that insulin levels correlate with placental size.^65^ Furthermore, maternal insulin promotes placental fatty acid transfer. The activation of insulin signaling pathways increases placental lipid carriers, which might increase fetal lipid transport and storage and even lower triglyceride levels in cord blood of the infant. Therefore, insulin resistance from the early stages of pregnancy alters both the placental structure and insulin signaling pathways in this tissue, resulting in fetal adiposity.^64^ Transplacental nutrient delivery to the growing fetus then promotes a rise in fetal insulin (Fig. 2). Fetal insulin assimilates glucose into cells, promoting fetal fat deposition, tissue accretion, glycogen storage, and growth.^18,21,37^ In response to the rise in glucose and fetal insulin, fetal leptin and IGF-1 production are stimulated.^18,49,51^ Fetal IGF-1 acts as a local growth modulator by initiating the differentiation and development of cells and causing fat deposition.^33,66^

HPL triggers hyperphagia of pregnancy to increase maternal fat deposition and to generate an easily accessible energy source for fetal growth. The increased food intake causes a rise in maternal leptin levels and is accompanied by hPL induced leptin resistance. hPL inhibits the transport of leptin across the blood–brain barrier, blunting its satiety-promoting effect on the hypothalamus, which creates a state of leptin resistance that allows the mother to maintain a high caloric intake despite fat accretion. This secures sufficient nutrient intake and availability to the fetus.^11,21,46,67^ The placenta also produces leptin. More than 95% of placental leptin is transported into the maternal circulation, where it contributes to the endocrine-mediated alterations in energy balance to increase the substrate availability through the mobilization of fat.^46,68^ A small amount of placental leptin crosses over into the fetal circulation where it mediates the anabolic actions of insulin, namely, protein and glycogen synthesis, tissue accretion, and fat storage.^21,63,69^ While leptin does not serve a satiety-promoting purpose in the fetus, it does signal sufficient nutrient availability, which increases the fetal metabolic rate, thus promoting mass accretion and growth. Fetal leptin production arises in the third trimester of pregnancy, stimulating cartilage and bone growth, and correlating with fetal fat mass.^16,68^

### Increased nutrient delivery

Multiple studies offer evidence for a local growth-modulating role of PGH, affecting the development and function of the placenta and therefore improving substrate delivery to the fetus.^34,67^ Placental function, development, and sufficient blood supply are essential to meet the metabolic demands of fetal growth.^11,18,22^ Recent studies have found that decreased levels of PGH are associated with late-onset small for gestational age (GA) fetuses that demonstrate histological lesions caused by placental under-perfusion, which is indicative of the association between PGH levels and placental (dys)function.^70,71^ Together with placental IGF-2, PGH regulates early trophoblast invasion and placental tissue differentiation. It promotes the adaptation of blood vessels supplying the placenta and the uterus, thereby improving substrate delivery to the fetus. Placental IGF-2 further contributes to increased nutrient delivery to the fetus, as it supports the simple and facilitated diffusion of substrates.^11,12,18,38^ Placental leptin also contributes to enhanced placental nutrient transfer through its interleukin-6-like proinflammatory qualities.^48^ The effect of PGH, hPL, and IGF-2 on enhancing substrate delivery demonstrates the impact of hormones on the materno-fetoplacental axis and their indirect regulation of fetal growth (Fig. 2).

### IGF production: direct and indirect effects on fetal growth

In addition to the modulation of maternal metabolism, PGH (as the main regulator) and hPL upregulate maternal IGF production (Fig. 2).^18,67^ Maternal IGF-1 promotes maternal tissue growth, improves uteroplacental blood flow, modulates maternal metabolism to ensure nutrient availability, and affects placental growth.^21,23^ Through the increase in substrate availability, maternal IGF-1 indirectly stimulates fetal growth. PGH and hPL also stimulate maternal IGF-2 production.^67^ Maternal IGF-2 presents a constitutive stimulus for fetoplacental growth through increased placental function and substrate transfer to the fetus.^37^ While maternal IGF-1 and IGF-2 both indirectly impact fetal growth similarly, IGF-1 acts predominantly on maternal substrate availability while IGF-2, with levels five to six times higher than IGF-1, mostly affects placental function and substrate delivery.^20^ In contrast to PGH, hPL is not only released into maternal circulation but also into fetal circulation, where it affects fetal IGF-1, IGF-2, and insulin production and therefore also directly contributes to fetal growth.^11^ In addition to the fetal production of IGF-2, the placenta functions as a major source for IGF-2. Placental release further increases the circulating levels of IGF-2 in the fetus. As IGF-2 stimulates an increase in fetal weight and fat mass via enhanced downstream effects of the IGF receptor and insulin receptor binding,^11,35,39^ a direct growth-promoting effect and a positive correlation between cord blood levels of IGF-2 and birth weight can be observed.^20,35^ Furthermore, IGF-2 also acts in concert with fetal prolactin to control adipose tissue differentiation, increasing fat mass, and adipocyte number.^54^ Fetal prolactin levels increase towards term in response to rising maternal estrogen levels and play a role in the maturation of preadipocytes and brown adipose tissue, which is responsible for the adaptation of postnatal thermogenesis.^24,52,72^ Rising estrogen levels also stimulate the maternal production of thyroid hormones. Maternal T3 and T4 partially cross the placenta and together with fetal T3 and T4 (which are first produced at a GA of 12 weeks) influence normal early fetal development and neurological maturation later in gestation.^13^

### Inhibition of growth

Placental CRH release stimulates the production of cortisol in the mother and the fetus. Corticosteroids play an important role in organ maturation and tissue specialization shortly before term, preparing the fetus for postnatal life. During pregnancy, fetal and maternal stress or inflammation induce a rise in cortisol levels, which suppresses IGF function and may lead to fetal growth restriction.^11,55^

### Hormonal influence on postnatal growth in term-born infants

**Fig. 3:**
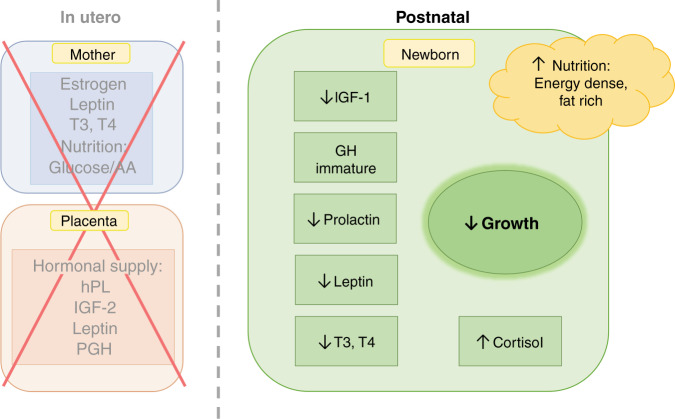
Hormonal homeostasis of the newborn and postnatal growth. The hormonal and nutritional withdrawal from the maternal-placental unit after birth leads to changes in hormonal homeostasis of the newborn and affects postnatal growth. AA amino acids, hPL human placental lactogen, IGF insulin-like growth factor, GH growth hormone, T3 triiodothyronine, T4 thyroxine.

Until birth at term, the fetus undergoes a growth process that is orchestrated by maternal–placental nutrition and GA-dependent hormonal control.^21^ When born, the glucose and amino acid-rich nutritional supply by mother and placenta ceases and a switch in nutrition to fat-rich breast milk or formula takes place (Fig. 3). This transition is accompanied by a negative energy balance during the first days of life.^43^ With the establishment of postnatal feeding, the increasing uptake of nutrients causes insulin levels to rise, allowing the infant to overcome the negative energy balance and initiate postnatal growth. Concurrently, leptin levels, which correlate with fat mass, normalize at 1 week of life.^51^

With the advancement of postnatal feeding, the nutritionally controlled rise in insulin levels directly triggers IGF-1 production in the newborn, which is responsible for postnatal growth and fat mass accretion.^35,51^ In parallel, a perinatal surge of thyroid-stimulating hormone (TSH) in the term-born infant triggers an increase in T3 and T4, which further stimulates growth and organ maturation postnatally.^13,58^

Once the GH-axis has fully matured, GH replaces the nutritionally controlled secretion of IGF and takes over the regulation.^14,27,28^ Until then, the infant experiences a relative IGF-1 deficiency caused by the immaturity of the GH-axis. In addition, slightly elevated cortisol levels postnatally and a change in the glucose threshold for insulin secretion contribute to a relative IGF-1 deficiency.^73^ The combination of this relative IGF-1 deficiency and the intake of energy-dense nutrition postnatally generates a physiological mismatch, in which the protein intake cannot be fully transformed into lean mass, which causes higher fat mass accretion compared to lean mass acquisition.^15,74,75^ While IGF-1 levels are relatively low at birth, they show a quick surge until they reach a plateau after 3 months, paralleling the postnatal rise in fat mass from 10 to 15% at birth to a plateau of 20–30%.^2,5,33^ In addition to fat mass accretion, length gain, bone accretion, growth rate, and chondrocyte proliferation in infants have been associated with IGF-1 levels that are mediated by nutritional intake, particularly protein intake.^32,33,56^ The loss of placental supply of IGF-2 and insufficient nutritional intake are responsible for lower IGF-2 levels postnatally.^24,35,76^ Although IGF-2 has less impact on postnatal growth than fetal growth in utero, it was found to be associated with adiposity in adult life.^35^

## Hormonal influence on postnatal growth in preterm infants

### General factors impacting the growth of preterm infants

For optimal postnatal growth to occur in preterm infants, various conditions must be met. These conditions include maturation of the neuroendocrine axis and target organs so that feedback mechanisms can reliably control growth, sufficient nutrient availability, and limitation of factors causing stress, such as comorbidities.^33^

It has been observed that preterm infants tend to be clinically stable during the first hours of life, also called the “honeymoon period.” However, after these first few hours, their condition often worsens.^77,78^ We hypothesize that the acute withdrawal of maternal and placental hormones largely contributes to this effect. Furthermore, the preterm infant copes with an energy deficit, because its energy requirements increase greatly (breathing, thermogenesis, organ function) compared to in utero and nutritional intake is insufficient.^43^ The premature withdrawal of the fetus from the uterus leaves the newborn with insufficiently developed organ systems. Therefore, respiratory support or ventilation is oftentimes required and can cause potential side effects.^79–81^ The early withdrawal of the fetus from the placental and maternal nutrient supply by preterm birth substantially disrupts infant nutritional support during the first weeks of life. The immaturity of the gut complicates early enteral feeding. Also, an immature capacity for oxidative phosphorylation delays the metabolic switch from glycolytic to more effective oxidative metabolism.^43,82^ These factors are associated with a high risk for cumulative nutritional deficits. Furthermore, prolonged parenteral nutrition, which is required for feeding advancement, increases the risk for catheter-related sepsis. Low birth weight and prematurity make infants more likely to need invasive treatments and procedures, further increasing the infant’s susceptibility to infections.^83–85^ Late-onset sepsis occurs in 10–40% of preterm infants (born <29 weeks).^86^ In those cases, doctors need to secure survival of these infants first before growth can be addressed. Therefore, the effect of disease on postnatal growth is tremendous. In addition to the disturbance of IGF, morbidity-related thyroid dysfunction and the immaturity of the thyroid system in preterm infants contributes to transient hypothyroxinemia of prematurity. Very low total levels of free T4 and normal to low levels of TSH can impair postnatal brain maturation and are associated with poor growth.^58^

### Hormonal impact on growth in preterm infants

Preterm birth disrupts the metabolic and endocrine materno-placento-fetal unit at a time when the preterm infant still has to undergo final organ maturation. Preterm birth implies serious vulnerability, low body weight, and lower body fat mass percentage (1–5% fat mass) compared with the term-born infant.^87^

**Fig. 4:**
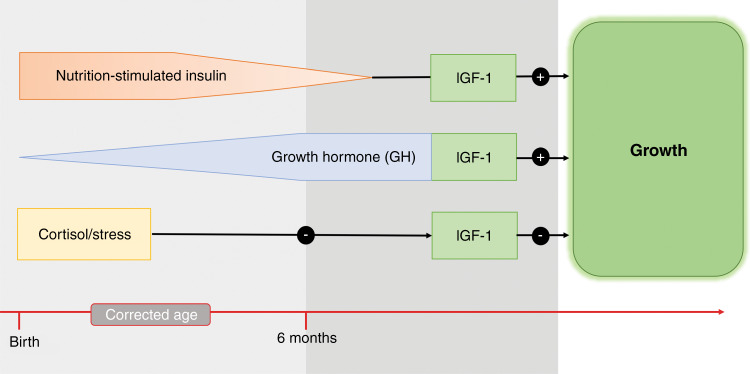
Postnatal growth control in newborns. The regulation of IGF-1 switches from nutrition to growth hormone control. IGF insulin-like growth factor.

In preterm infants, the immaturity of organ systems is associated with the insufficient secretion of hormones. The premature withdrawal from the maternal and placental hormone supply leads to decreased hormonal levels, which meet a low number of receptors, rendering hormonal action even more ineffective.^24,59^ During gestation, IGF-2 stimulates adipocyte growth and mass accumulation by binding to the insulin and IGF receptors and enhancing their downstream effects.^35^ After birth, growth is predominantly regulated by IGF-1.^35^ In preterm infants, the placental secretion of IGF-2 ceases prematurely, and additionally the postnatal IGF-1 secretion cannot fully compensate for the lack of intrauterine IGF-2-mediated mass accumulation. This is especially true for preterm infants born at extremely low GAs (<28 weeks), leading to high risk for poor general growth, poor brain growth, and neonatal morbidities such as intraventricular hemorrhage, retinopathy of prematurity (ROP), bronchopulmonary dysplasia (BPD), and necrotizing enterocolitis (NEC).^43^ In very preterm infants, the regulation of thyroid hormones is still immature. This hypothalamic immaturity is indicated by a reduced TSH response to extrauterine exposure.^24^ In addition, premature birth abruptly terminates the maternal contribution of thyroid hormones and prohibits the perinatal rise in TSH, explaining low levels of T4 in the preterm-born infants. This can lead to transient hypothyroxinemia, which impairs optimal postnatal growth and brain maturation.^13,58^ However, neonatal hypothyroidism is uncommon, as thyroid hormone levels are closely monitored and supplemented if needed.^13,88^ Furthermore, preterm infants are also leptin-deficient beyond term gestation. Leptin increases the basal metabolic rate by promoting mass accretion when sufficient nutritional supply is available; it mediates the anabolic actions of insulin, stimulates bone growth, and exhibits interleukin-6 like proinflammatory qualities.^26,48,49^ Therefore, the leptin deficiency in preterm infants impairs sufficient postnatal growth and renders preterm infants susceptible to infections and permanent metabolic, neuroendocrine, and developmental problems.^89,90^ Insulin activity in preterm-born infants is also lower than in term-born infants.^91^ Insulin stimulates fat storage and glycogen synthesis; therefore, immature enzyme activity in preterm infants limits the metabolism of nutrients after birth, which results in insufficient nutritional intake.^11,56,92^ Postnatal growth depends on nutritionally stimulated insulin release, which then stimulates the IGF-1 production and release (Fig. 4). Low insulin activity and consequently low levels of IGF-1, which increases glucose and amino acid uptake to promote growth, therefore, lead to initially restricted postnatal growth in preterm-born infants.^10,34,36^ Unlike term-born infants, whose IGF-1 levels start to increase rapidly after birth, preterm-born infants experience a period of perinatal growth restriction, throughout which IGF-1 levels stay low and only rise slowly.^43,74,75^ Once preterm-born infants have adapted to the extrauterine environment, they can metabolize nutrients effectively. In addition, improved health and organ maturation further contribute to optimal growth conditions, which leads to a turning point from growth restriction to growth acceleration. Consequently, the increasing nutrient intake and rise in insulin stimulate the production of IGF-1 in preterm infants.^28,56^ However, the IGF-1 levels in preterm infants remain lower than in utero levels at corresponding ages.^40^ As a result, it could be hypothesized that the relatively lower IGF-1 levels in preterm-born infants lead to altered postnatal growth patterns, including excessive accumulation of fat mass.^28^ The stimulation of adipogenesis rather than lean mass accretion is possibly mediated by a mismatch of relatively low levels of IGF-1 along with sufficient nutrient intake and insulin secretion. This is also described by Yumani et al. who observed that states of low IGF-1 levels stimulate the growth of adipose tissue.^33^ This mismatch can be further amplified by elevated cortisol levels in preterm infants due to neonatal morbidities and perinatal stress.^55^ Cortisol limits IGF-1 secretion and contributes to elevated fat mass accretion (Fig. 4). For proper lean mass accretion, sufficient IGF-1 levels and protein intake (nutrition) are essential.^33,93^ As both are limited in preterm infants, the accretion of lean mass during the postnatal period is restricted.

### Fetal and postnatal development in the physiological context

The differences between birth at term and preterm birth hugely affect the development of postnatal body composition. Until birth at term, the aim of physiological fetal growth is the maturation of brain, organ, and muscle tissue. At term, the fetus will be mature and relatively lean, allowing passage through the narrow maternal birth canal. Postnatally, the adaptation to extrauterine life is accompanied by fat mass accretion, which is needed for thermoregulation, energy storage, and infection prevention.^94,95^

At preterm birth, brain and muscle tissue have not fully matured.^96^ Therefore, these infants require optimal nutrition to grow brain, organ, and muscle tissue. Furthermore, their postnatal adaptation to extrauterine conditions through fat mass accretion, while similar to term-born infants, occurs at younger PMAs.^3^ This explains the higher levels of fat mass observed in preterm-born infants at term age compared to their term-born counterparts.

### Factors improving growth in preterm infants

Preterm infants are exposed to metabolic-endocrine disruption. Low levels of early postnatal IGF-1 seem to be the root of the problem for suboptimal body composition in preterm-born infants, and so an early increase in nutrition-mediated IGF-1 is postulated to be the solution.^33^ Particularly, low intake of dietary protein is associated with low IGF-1 levels. The state of low IGF-1 levels and subsequent postnatal growth restriction, in turn, are associated with a tendency for fat mass accretion in childhood.^33,82^ Early increases in protein intake enhance postnatal IGF-1 production through improved insulin release and lead to a favorable metabolic programming with increased levels of lean mass.^33,56,93^ Lafeber et al. also found that immediate postnatal high-protein nutrition limits postnatal growth restriction and consequently prevents the need for exaggerated fat mass accumulation. In preterm infants with neonatal intensive care-acquired nutritional deficits, optimized protein intake through post-discharge nutrition limited fat mass deposition. This further displays the effect of optimized protein intake on preterm body composition and on the risk of metabolic diseases in adulthood.^82^ The risk of neonatal morbidities such as ROP was also reduced with higher protein intake. Increased protein intake impacts ROP risk by mediating increases in IGF-1 levels, which is needed for maximum activation of the vascular endothelial growth factor for vascular endothelial cell proliferation, resulting in a reduced risk for ROP.^56,97,98^

## Critical appraisal of current intervention studies and considerations to improve the growth of preterm infants

### Intervention with GHs

In several studies, preterm and very preterm infants underwent GH treatment aimed at improving postnatal growth and body composition and reducing the risk of adult metabolic disease. According to de Kort et al., the percent body fat corrected for age measured in preterm infants decreased in response to GH treatment, while term-born infants showed no reduction. Hence, the lipolytic effect of GH in preterm adipose tissue is greater than in term-born infants.^91^ Although percent body fat decreased in preterms, improving their body composition, their lean body mass values remained low (*n* = 143, GA: <36 weeks). Huysman et al. provided GH treatments to very preterm infants (*n* = 30, GA: <32weeks) who underwent BPD-induced glucocorticoid therapies for weaning from mechanical ventilation. GH treatment was unable to prevent the growth impairment caused by the high-dose glucocorticoids.^99^

The treatment with GH might have limitations that arise from immaturity and the limited number of GHRs in preterm-born infants.^24,59^ Furthermore, the study lacks data regarding the long-term effects of the treatment with GH. As GH exerts its effect via GHRs that further stimulate the release of IGF-1, the impact on growth in preterm-born infants might be minimized.

### Intervention with IGF-1

Poor brain development, disturbed retinal vascularization, and restricted growth are widely agreed to be caused by reduced IGF-1 release in preterm infants due to nutritional deficiency and immaturity.^75^ Common complications of prematurity, such as ROP, BPD, and NEC, are also associated with low IGF-1 levels.^40^ Hansen-Pupp et al. and Hellström et al. reported a correlation between the level of IGF-1 at birth and the risk of ROP, while the severity of ROP was correlated with the duration of low IGF-1 levels.^75,100^ Further highlighting the importance of IGF-1, a 2.2-fold increase in the relative risk for morbidity in preterms has been described when IGF-1 levels at 33 weeks PMA measure <33 µg/L.^100^ Experimental and clinical studies indicate that early IGF-1 supplementation improves growth in catabolic states.^28^ Hansen-Pupp and Ley demonstrate that the continuous longitudinal intravenous infusion of recombinant human IGF-1 and IGFBP-3 (rhIGF-1/rhIGFBP-3) increases mean IGF-1 levels in preterm infants, reducing the incidence of BPD, ROP, and other associated morbidities. A study by Ley showed that reference levels of IGF-1 can be achieved by IGF-1 supplementation (*n* = 5, GA: 26–27 + 2/7 weeks).^101^ This effect was confirmed by Hansen-Pupp et al., who also reported higher mean IGF-1 serum levels in treated infants (23 µg/L) compared to untreated infants (14 µg/L) (*n* = 9 versus *n* = 10, GA: 23–27 + 6/7 weeks). In addition, a positive trend in the reduction of BPD was observed in the treated group (44%) compared to the control group (70%).^75^ In a recent phase 2 trial with rhIGF-1/rhIGFBP-3 supplementation, a decreased occurrence of severe BPD in preterm infants (*n* = 61, GA: 23–27 + 6/7 weeks) was also confirmed.^102^

In summary, recent studies using IGF-1 show a trend towards improved growth and reduced neonatal morbidities in preterm infants. Limitations arise from small sample sizes and a lack of long-term data regarding the treatment with IGF-1. However, considering the premature withdrawal of PGH, placental IGF-2, and maternal hormone supply that occurs with preterm birth, as well as the physiologically lower IGF-1 levels in preterm infants, the optimization of IGF-1 levels seems to be a promising approach for the improvement of preterm growth and development.

### Physiological adaptation of body composition in preterm and term infants: a hypothesis

During a healthy pregnancy, the hormone and nutrient supply to the fetus is provided by the maternal–placental unit. During the third trimester, the fetus matures for postnatal life. A rise in fetal prolactin levels at 28–30 weeks parallels an elevation in maternal estrogen levels and contributes to fetal fat mass accretion.^24^ Concurrently, levels of leptin, fetal IGF-1, partially stimulated by hPL, and placental IGF-2 continue to rise.^20,103^ However, with birth, the newborn faces an endocrinologic withdrawal from the maternal–placental unit. The sudden lack of hormonal supply (e.g., leptin, IGF-2, hPL, estrogen) requires postnatal adaptation of the maturing GH-axis in the fetus to take over the control of postnatal growth. However, immaturity of the GH-axis has been observed until ~6–9 months of life.^14,24,27^ Until then, the nutrition-driven pathway of insulin stimulating IGF-1 secretion controls growth (Fig. 4).^20^ This is reflected by a coinciding increase in fat mass that usually plateaus by 2–6 months after birth.^104^ The increase in fat mass could be seen as an adaptive response to birth that leads to the achievement of optimal amounts of fat mass required for extrauterine life.^3^ The following hypothetical mechanisms for an increase in fat mass can be proposed. In fetuses that stay in utero until term, increasing levels of growth factors such as prolactin, IGFs, and leptin during the third trimester stimulate fetal fat mass accretion, which can be seen in the data of the reference fetus.^87^ Sufficient leptin levels act as a switch to increase the metabolic rate, as leptin levels correlate positively with the resting energy expenditure.^47^ This leads to increased utilization of the nutritional energy, resulting in mass accretion. Postnatally, the nutritional intake through breast milk provides greater amounts of fat (fat intake: 5–7 g/kg/day) to meet the energy demands of extrauterine life.^105,106^ A possible mismatch between the relatively low IGF levels during the first month of life and the sufficient nutritional intake might lead to favored fat mass accretion instead of muscle and organ development. This likely causes accelerated fat deposition. Relatively low levels of IGF are provoked by the maturing GH-axis and less responsive IGF production to the nutrient-insulin pathway.

In preterm infants, levels of growth-controlling hormones are reduced due to the following factors: early removal from the placental hormonal supply, intensive medical care, and nutritional deficiencies and neonatal morbidities during the first weeks of life. These clinical conditions increase the stress-related cortisol release, which in addition to an immature GH-axis, and prohibits IGF production, which leads to growth restrictions.^11^ After normalizing these clinical conditions, sufficient amounts of nutritional supply will be taken up. However, GH levels are still inadequate. Insufficient leptin levels leave the infant with a low metabolic rate, despite energy uptake, and reduced host defense response to pathogens, as leptin has emerged as an important modulator of immune function.^50^ In addition, relatively low IGF levels might induce fast fat deposition instead of lean mass accretion at early PMAs. Compared to term infants, the postnatal adaptation of fat mass occurs in preterm infants at earlier PMAs, resulting in body fat percentages of 20–30 % at term age.^2^ By the age of 4–6 months postnatally, absolute fat mass is no longer different between preterm and term-born infants.^2,107,108^ With the maturation of the GH-axis, IGF-1 levels then reach sufficient levels, and lean mass accretion instead of fat mass deposition is favored. This supports that the fast accretion of fat mass in preterm infants postnatally is part of an adaptive response to birth.

### Perinatal metabolic-endocrine disruption: an unrecognized perinatal disorder

Over the past two decades, neonatologists have primarily focused on the survival of neonates and have been successful in improving this. Postnatal intensive care targets the treatment of acute life-threatening conditions such as respiratory distress syndrome, hypotension, hypoglycemia, patent ductus arteriosus, and sepsis. Today, the treatment of these morbidities is generally successful and has resulted in a reduction in mortality rates. The improved survival rates allow for optimization of the quality of survival through the introduction of less invasive techniques, personalized medicine, and improved nutrition for the achievement of desired neurodevelopmental outcomes and reduced risk for chronic diseases in adulthood. However, the perinatal metabolic-endocrine disruption caused by the withdrawal of hormonal and metabolic supply from the maternal–placental unit remains an often-unrecognized perinatal disorder. It is reflected in the “honeymoon period” and subsequent worsening of clinical conditions observed in preterm infants after the withdrawal of maternal–placental hormones. The clinical picture is similar to panhypopituitarism, along with the increased release of stress hormones. This condition is outside the scope of the usual clinical focus, as the effects of metabolic-endocrine disruption are initially hidden. However, it seems to be related to adverse metabolic programming and affects growth, body composition, and neonatal outcomes such as ROP, BPD, and neurodevelopment. Further, reference values for clinical chemistry obtained from preterm infants, which do reflect the normal physiology of a fetus in utero, are likely confounded and prevent neonatologists from recognizing hormonal deficiencies. Therefore, considering DOHaD, perinatal metabolic-endocrine disruptions should be considered a perinatal disorder, and their treatment should be of importance in further research.

## Conclusion

Birth disconnects the infant from the maternal–placental unit. This dissociation of the unit of mother, placenta, and fetus is physiological in term infants, but leads to multiple endocrine and nutritional disruptions in immature preterm infants. The withdrawal of maternal–placental hormones and nutrients results in a clinical picture similar to a “panhypopituitarism.” This withdrawal affects hormones such as IGF-1, leptin, thyroid hormones, steroids, and estrogen and could result in growth impairments, a body composition deviating from term-born infants, and unfavorable neonatal outcomes. Appropriate and GA-adapted nutrition is the prerequisite to achieve optimal growth in preterm infants. However, ideal nutrition alone seems to be insufficient. Research on metabolic-endocrine disruption and the pathophysiology of involved hormones (tissue, thyroid, adrenal, pancreatic, pituitary, and placental hormones) is needed as a basis for therapeutic approaches. The supplementation of the growth-promoting hormone IGF-1 in preterm-born infants, and thyroid hormones in very preterm infants could be beneficial in preventing growth-related disorders and enhancing the best possible development. Short- and long-term implications of therapies need to be tested in animal models and clinical trials.
